# Aortic valve repair with annuloplasty

**DOI:** 10.1093/icvts/ivaf146

**Published:** 2025-07-02

**Authors:** Francesco Giosuè Irace, Ilaria Chirichilli, Raffaele Scaffa, Chiara Bellome, Mario Torre, Andrea Salica, Giulio Folino, Ruggero De Paulis

**Affiliations:** Department of Cardiac Surgery and Heart Transplantation, San Camillo Forlanini Hospital, Rome, Italy; Department of Cardiac Surgery and Heart Transplantation, San Camillo Forlanini Hospital, Rome, Italy; Department of Cardiac Surgery, European Hospital, Rome, Italy; UniCamillus, International University of Health Sciences, Rome, Italy; Department of Cardiac Surgery, Ospedale Ruggi e D’ Aragona, Salerno, Italy; Department of Cardiac Surgery, European Hospital, Rome, Italy; Department of Cardiac Surgery, European Hospital, Rome, Italy; Department of Cardiac Surgery, European Hospital, Rome, Italy; UniCamillus, International University of Health Sciences, Rome, Italy

**Keywords:** aortic root, aortic valve, aortic valve repair, aortic annulus, aortic regurgitation

## Abstract

**OBJECTIVES:**

Aortic valve repair procedures for aortic valve regurgitation have been progressively adopted in the last decades. We analysed our results with an external ring annuloplasty and/or leaflet repair.

**METHODS:**

From April 2014 to December 2023, 61 consecutive patients underwent aortic valve repair with external Teflon ring annuloplasty. The external ring was made of an 8–9 mm Teflon strip, to reduce the annulus diameter between 21 and 23 mm. Cusp effective height (eH) was assessed with a caliper (not used before 2018), and any cusp prolapse was corrected by free margin plication, to obtain a 9–10 mm eH for all cusps.

**RESULTS:**

Patients (72.1%) had severe aortic regurgitation (AR), and associated supracoronary aneurysm repair was performed in 42.6%. No operative death occurred; residual AR more-than-moderate was present in one patient only. The 8-year overall survival was 97.4 ± 2.6%, freedom from endocarditis 98.3 ± 1.7% and freedom from thromboembolism 100%. Recurrence of severe AR with need for reoperation was predicted by the presence of a particularly enlarged aortic annulus (≥28 mm, *P* < 0.01) and the non-routinary use of cusp caliper (*P* = 0.03).

**CONCLUSIONS:**

The external Teflon ring annuloplasty appears a safe procedure with high overall survival, freedom from endocarditis and freedom from thromboembolism at 10 years. Recurrence of severe AR could be related to patient selection and learning curve.

## GRAPHICAL ABSTRACT

**Figure:**
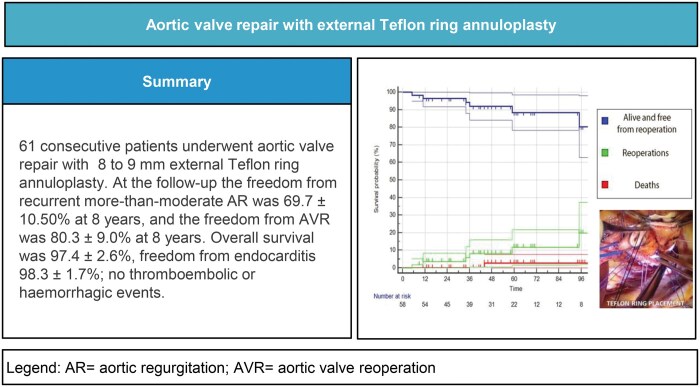

## INTRODUCTION

In the past 20 years, reconstructive techniques for addressing aortic regurgitation (AR), whether accompanied by an aortic root aneurysm or not, have emerged as a viable and increasingly popular alternative to valve replacement [1, 2]. Achieving a successful and lasting aortic valve repair leads to a low rate of valve-related complications and potentially enhances patient survival [3, 4]. The primary goal in aortic valve repair is to re-establish a normal valve geometry, as the stability of aortic valve repair can be significantly enhanced by employing an anatomic-oriented approach [5, 6].

Annular dilatation is a common feature in repairable AR, especially in bicuspid aortic valves (BAV), even without dilatative aortopathy. An annular diameter exceeding 25 mm is generally considered dilated [7] and in need of correction during repair surgery. Stabilizing or reducing the aortic annulus has been shown to significantly enhance the durability of BAV repair [8, 9].

The significance of aortic annulus dilatation in the development of chronic AR was highlighted as early as 1958 by Taylor and colleagues when they documented their experience with circumclusion of the aortic root [10]. Since that time, annuloplasty has become a crucial element of aortic valve repair, leading to the proposal of various techniques.

Various groups have utilized sub-commissural annuloplasty due to its simplicity [11, 12].

However, postoperative progressive annular dilatation has been noted, leading to repair failures and a consequent abandonment of this technique.

Another approach involves an internal, rigid ring with an elliptical shape, designed to be implanted below the cusp insertion with extensions below the commissures. Although several series with limited numbers and follow-ups have been published, early failures were linked to cusp tissue abrasion or ring dehiscence [13]. Finally, a suture annuloplasty placed at the basal level was reintroduced in a modified form in 2009 to fit the ventricle-arterial junction in the sinus and reduce external dissection. Positive results and annuloplasty stability have been reported [14].

On the other side, there is the external approach where a flexible ring is positioned externally around the anulus and it is fixed by a series of sutures positioned below the leaflet inside out of the aortic annulus in a manner similar to that used for the reimplantation technique [15–18].

Aortic valve annuloplasty is the primary step in isolated BAV repair for normalizing the functional annulus, or virtual basal ring (VBR), of the aortic root. However, annuloplasty alone rarely completely restores aortic valve shape and function; adjunctive cusp repair is often necessary. The benefit of sub-commissural sutures is debatable, and comparing the results of other techniques is challenging due to uncontrolled confounding variables and inconsistent end-points in the literature. Currently, it is not possible to provide clear recommendations on which technique is best suited for specific pathologies. Future studies should control for confounding factors and include follow-ups of at least 5 years to provide more definitive guidance.

Since 2014, the European Hospital has standardized the use of aortic annuloplasty to treat patients with AR due to annulus dilatation. This study aims to describe and analyse the mid-term outcomes of aortic valve repair using external Teflon ring annuloplasty, with emphasis on predictors of early and late failure.

## MATERIALS AND METHODS

### Ethics statement

The study was approved by the European Hospital Ethical Committee (No. IRB 2021/01 on 15 January 2021). Informed consent was waived because of the retrospective nature of the study with anonymous clinical data analysis.

This is a single-centre observational cohort study. From April 2014 to April 2023, 61 consecutive, unselected patients affected by aortic valve regurgitation (isolated or in association with other cardiac conditions) underwent an aortic valve repair procedure at European Hospital of Rome, by a single surgeon. All patients received preoperative echocardiography and ECG-gated CT scan, and, when appropriate, coronary angiography. Patients with mixed aortic valve pathology (stenosis + regurgitation) were excluded from the repair procedure.

Early outcomes in terms of operative death, major postoperative complications (stroke, atrioventricular block, surgical haemostasis revision, ICU length of stay) and predischarge echocardiography were evaluated. Mid-term follow-up was performed through telephonic interview and/or outpatient clinic evaluation, follow-up echocardiography was performed when possible at our echocardiography laboratory or was acquired from trusted referral cardiologists, follow-up was completed on 31 March 2024. Outcomes were evaluated in terms of overall survival, cardiac-related survival, freedom from reoperation, freedom from recurrent AR, incidence of infective endocarditis and thromboembolic or haemorrhagic complications.

### Surgical technique

**Figure 1:**
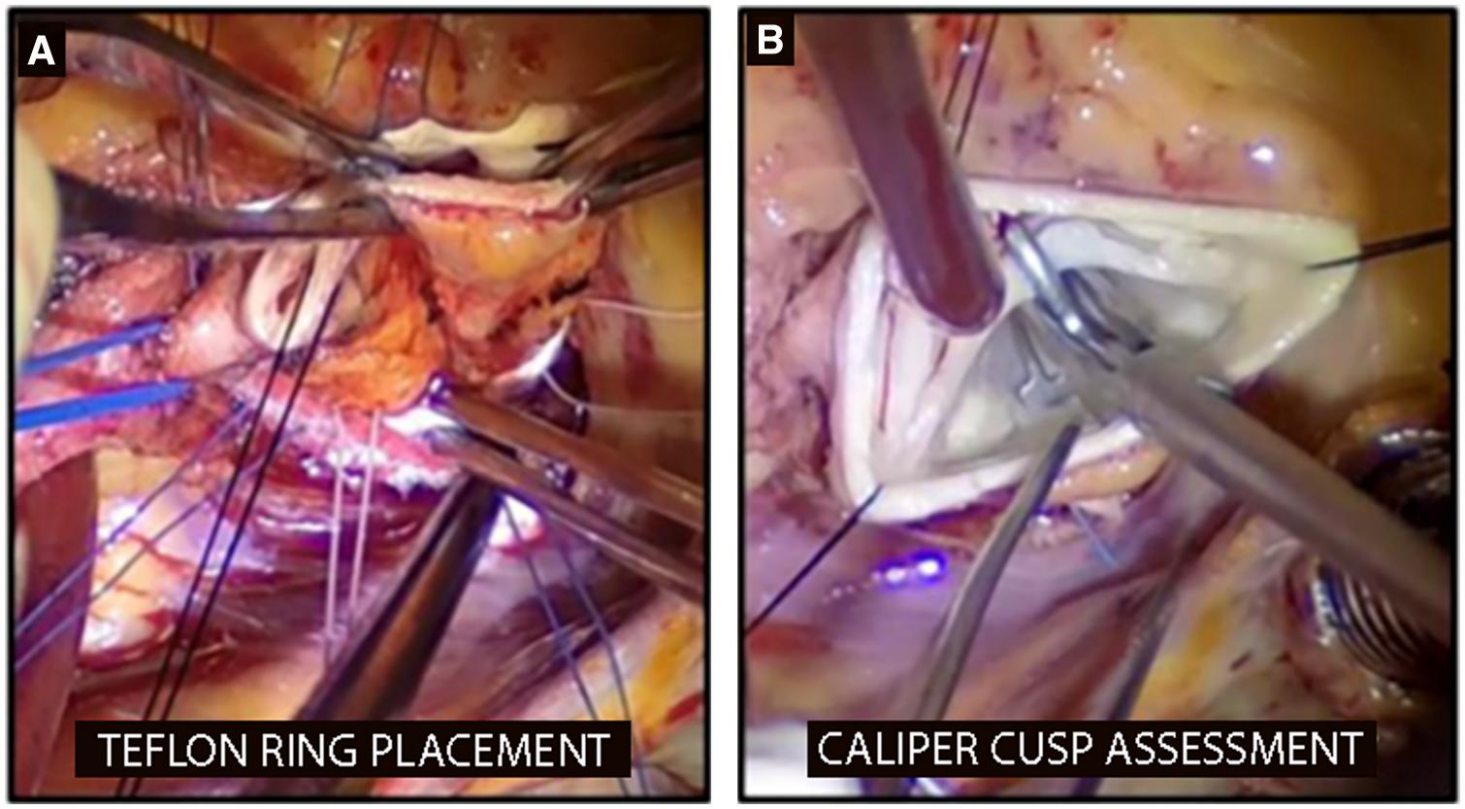
(**A**) Annuloplasty Teflon strip placement. (**B**) Cusp effective height assessment.

The aortic valve repair technique used in our centre has been already described in a previous study [19]. In brief, the aorta is transected at the level of the sino-tubular junction (STJ); deep dissection of the root is performed as for a reimplantation procedure. Between 6 and 9 pledgeted sutures are placed from inside out at the level of the VBR, avoiding any distortion or interference with the normal movement of the leaflets; to reduce the risk of atrioventricular block, the suture below the right/non-coronary commissure is spaced slightly wider than the others. Then the external ring for annuloplasty is made with a Teflon strip (5–7 mm in width), its length is tailored to reduce the annulus to a diameter between 21 and 23 mm and to re-establish a 1:1.3 ratio with the STJ diameter (Fig. 1A), when indicated the ascending aorta was replaced with Dacron graft (26–30 mm) in order to re-establish the correct ratio. The strip is placed outside the aortic root base, passing it beneath the coronary ostia. The sub-annular sutures are then passed at the appropriate level through the Teflon strip and the sutures are tightened on a Hegar’s dilator positioned across the aortic valve, to avoid excessive annular reduction. The Hegar’s dilator size is chosen according to the desired annular dimensions (usually between 21 and 23 mm). After annuloplasty, leaflet assessment is performed: the cusp effective height is assessed with a caliper and any cusp prolapse is corrected by free margin plication with 5/0 polypropylene suture, in order to obtain a 9–10 mm effective height (Fig. 1B). If necessary, the free margins are shaved and raphes (in bicuspid valves) are released. Then, the transected ascending aorta is sutured back at the ST junction, or, in the presence of ascending aorta aneurysm, a standard supracoronary ascending aorta replacement is performed.

### Statistical analysis

The processing of all data was conducted by an independent statistician utilizing an IBM-SPSS 26.0 (Armonk, NY) and R Statistical Software (v4.1.3; R Core Team 2021) workstation. Continuous variables are reported as the mean and standard deviation, following the testing of normality with the Kolmogorov–Smirnov methodology. Non-normally distributed data are reported as the median and interquartile range. Categorical variables are reported as the absolute number and relative incidence. The Kaplan–Meier estimator was used to estimate the survival probabilities over time for the cohort. The survival curves were compared between treatment groups using the Log-Rank test. Confidence intervals at 95% were calculated for each survival estimate, and Kaplan–Meier curves were plotted to visualize the survival distribution. Restricted mean survival time analysis was performed to compare groups with different follow-up times. As this is a single-centre observational cohort study, it was beneficial to configure the manuscript according to the STROBE checklist (Supplemental Material S1). The statistical analyses are in accordance with the “Statistical and data reporting guidelines for the European Journal of Cardio-Thoracic Surgery and the Interactive CardioVascular and Thoracic Surgery” [20]. Kaplan–Meier curves have been elaborated in accordance with the guidelines.

## RESULTS

### Baseline patients’ characteristics

Sixty-one patients were enrolled in our study; they were predominantly males (98.4%), mean age was 44years old (13). The preoperative echocardiography showed the presence of BAV in 55 patients (90.1%), of whom 39 (63.9%) had a 3-sinusesfused bicuspid valve and 16 (26.2%) a 2-sinuses bicuspid valve. Preoperative LVEF was 58 (7) %, mean left ventricle end-diastolic diameter was 61 (12) mm. The aortic valve regurgitation was defined according with the current European guidelines [21]: 72.1% had severe AR, 26.2% moderate AR, 1.6% mild AR; a regurgitant eccentric jet was present in 54 patients (88.5%); in patients with non-severe AR, the surgical indication was driven by the presence of concomitant conditions. Mean aortic diameters were as follows: annulus 27 (3) mm, root 40 (4) mm, STJ 34 (5) mm, tubular ascending aorta 42 (7) mm. Baseline patients characteristics are summarized in Table 1. Two patients had a previous cardiac surgery.

**Table 1:** Preoperative clinical and echocardiographic characteristics^a^

No of patients	61
Male sex	60 (98.4%)
Age, *y*	44 (13)
Body surface area, *m^2^*	1.95 (0.17)
**Bicuspid aortic valve**	55 (90.1%)
*Fused*	39 (63.9%)
Left-right	35 (57.4%)
Right non-coronary	4 (6.5%)
Left non-coronary	0 (0)
*Two-sinuses*	16 (26.2%)
**Echocardiographic findings**	
LVEF, *%*	58 (7)
LVEDD, mm	61 (12)
LVESD, mm	42 (5)
LVEDV, ml	182 (44)
LVESV, ml	75 (24)
Mitral regurgitation > 2+	3 (5%)
Aortic regurgitation, none	0
Aortic regurgitation, trivial	0
Aortic regurgitation, mild	1 (1.6%)
Aortic regurgitation, moderate	16 (26.2%)
Aortic regurgitation, severe	44 (72.1%)
Aortic regurgitation with eccentric jet	54 (88.5%)
Aortic annulus diameter, mm	27 (3)
Aortic root diameter, mm	40 (4)
Sino-tubular junction diameter, mm	34 (5)
Ascending aorta diameter, mm	42 (7)

a Values are presented as mean (standard deviation), or frequencies (%).

LVEDD: left ventricle end-diastolic diameter: LVEDS: left ventricle end-systolic diameter; LVEDV: left ventricle end-diastolic volume; LVEF: left ventricle ejection fraction; LVESV: left ventricle end-systolic volume.

### Operative and early outcomes

Associated ascending aorta replacement for supracoronary aortic aneurysm was performed in 26 patients (42.6%), other cardiac procedures were performed in 5 patients (8.2%). Mean CPB time was 90 (26) min, and mean cross-clamp time was 72 (12) min. There were no operative (30 days) deaths. We had one case of postoperative ischaemic stroke (1.6%) and two patients who necessitated permanent pacemaker implant for high-degree atrioventricular block (3.3%). Postoperative, predischarge echocardiography showed the presence of more-than-mild residual AR in only one patient (1.7%); the mean and peak AV pressure gradient were, respectively, 11 (4) mmHg and 18 (7) mmHg. The operative and early outcomes are reported in Tables 2 and 3.

**Table 2:** Operative data^a^

No of patients	61
Previous cardiac surgery	2 (3.3 %)
CBP time, min	90 (26)
X Clamp time, min	72 (12)
Associated ascending aorta replacement	26 (42.6%)
**Graft size**	
26 mm	2 (3.3%)
28 mm	8 (13.1%)
30 mm	16 (26.2%)
Associated other cardiac surgery	5 (8.2%)

a Values are presented as mean (standard deviation) or frequencies (%).

CPB: cardiopulmonary bypass.

**Table 3:** Early outcomes^a^

No of patients	61
Operative death (30 days)	0 (0)
Stroke	1 (1.6%)
Haemostasis revision	7 (11.4%)
PM implant	2 (3.3%)
Paroxysmal AF	13 (21.3%)
ICU stay, days	1.9 (1)
**Postoperative echocardiographic findings**	
Residual AR, none	42 (70%)
Residual AR, mild	17 (28.3%)
Residual AR, moderate or worse	1 (1.7%)
LVEF, %	54 (7)
Mean aortic valve gradient, mmHg	11 (4)
Peak aortic valve gradient, mmHg	18 (7)

a Values are presented as mean (standard deviation) or frequencies (%).

AF: atrial fibrillation; AR: aortic regurgitation; ICU: intensive care unit; LVEF: left ventricle ejection fraction; PM: pacemaker.

### Follow-up

**Figure 2:**
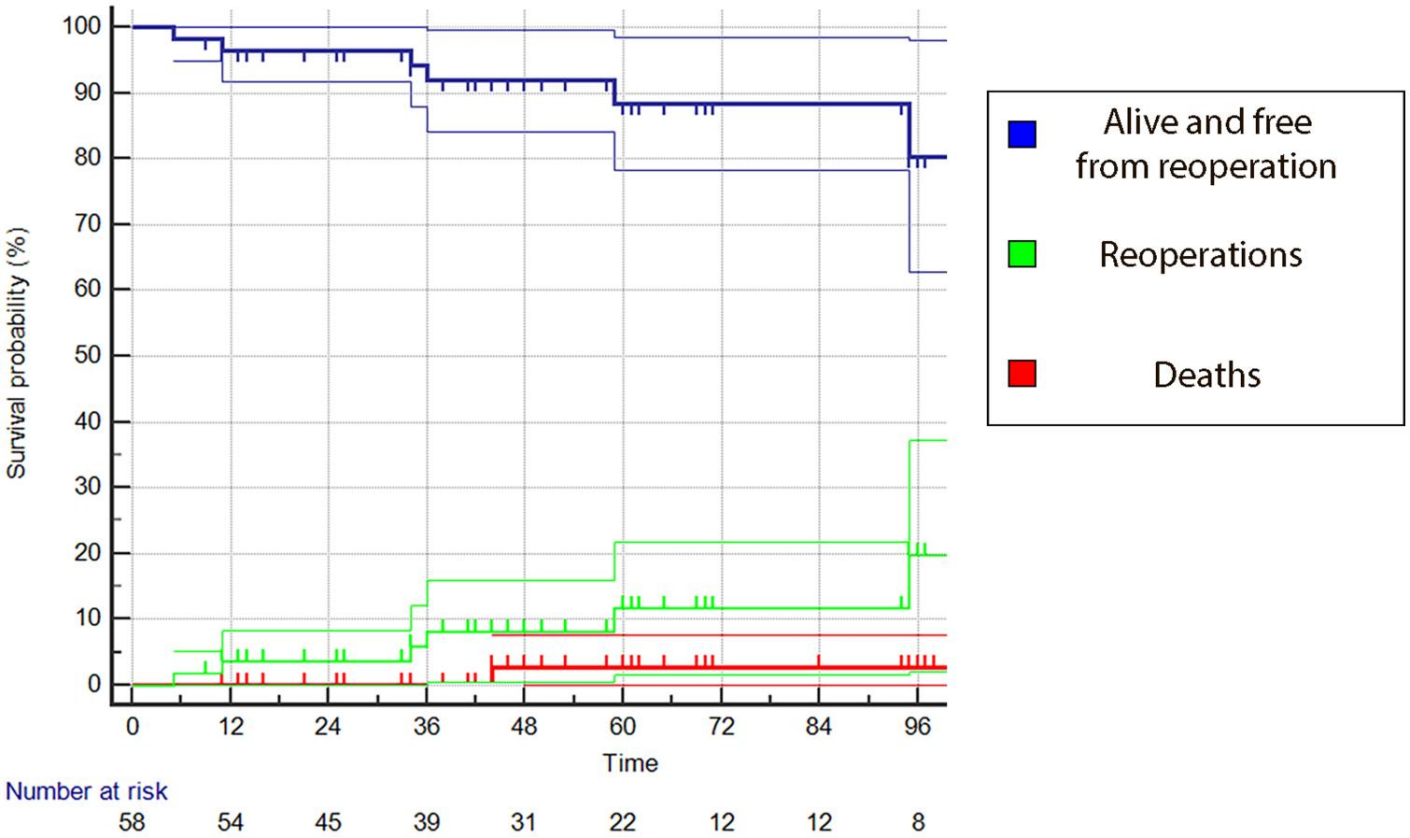
Combined Kaplan–Meier curve showing overall survival + freedom from reoperation, reoperation incidence and death incidence.

Follow-up index was 0.93, with three patients lost; the mean follow-up time was 57 (31) months (4.75 (2.6) years), while the mean echocardiographic follow-up was 47 (31) months. We had one death after 9 months for complicated pneumonia: overall survival was 97.4 ± 2.6% at 4 and 8 years, freedom from cardiac death was 100%. One case of infective endocarditis occurred 5 months after operation and resulted in root pseudoaneurysm and severe AR: the patient then received a mechanical prosthetic valve. We found seven more recurrent AR (moderate or worse), for a total of eight patients (13%); AR recurrence occurred at a mean follow-up of 3.7 (2.7) years. Six out of eight underwent reoperation with aortic valve replacement. As a consequence the freedom from recurrent more-than-moderate AR was 92.1 ± 3.8% at 4 and 69.7 ± 10.50% at 8 years, and the freedom from aortic valve reoperation was 91.9 ± 3.9% at 4 and 80.3 ± 9.0% at 8 years (Fig. 2). There was no significant difference in mean and peak aortic valve gradients between the patients with recurrent AR and those without recurrence (mean PG 9.3 (3.8) mmHg vs 10.8 (4.2), *P*=0.38; peak PG 15.2 vs 18.8 (7.1) mmHg, *P*= 0.24).

We had no thromboembolic or haemorrhagic complications reported.

**Figure 3:**
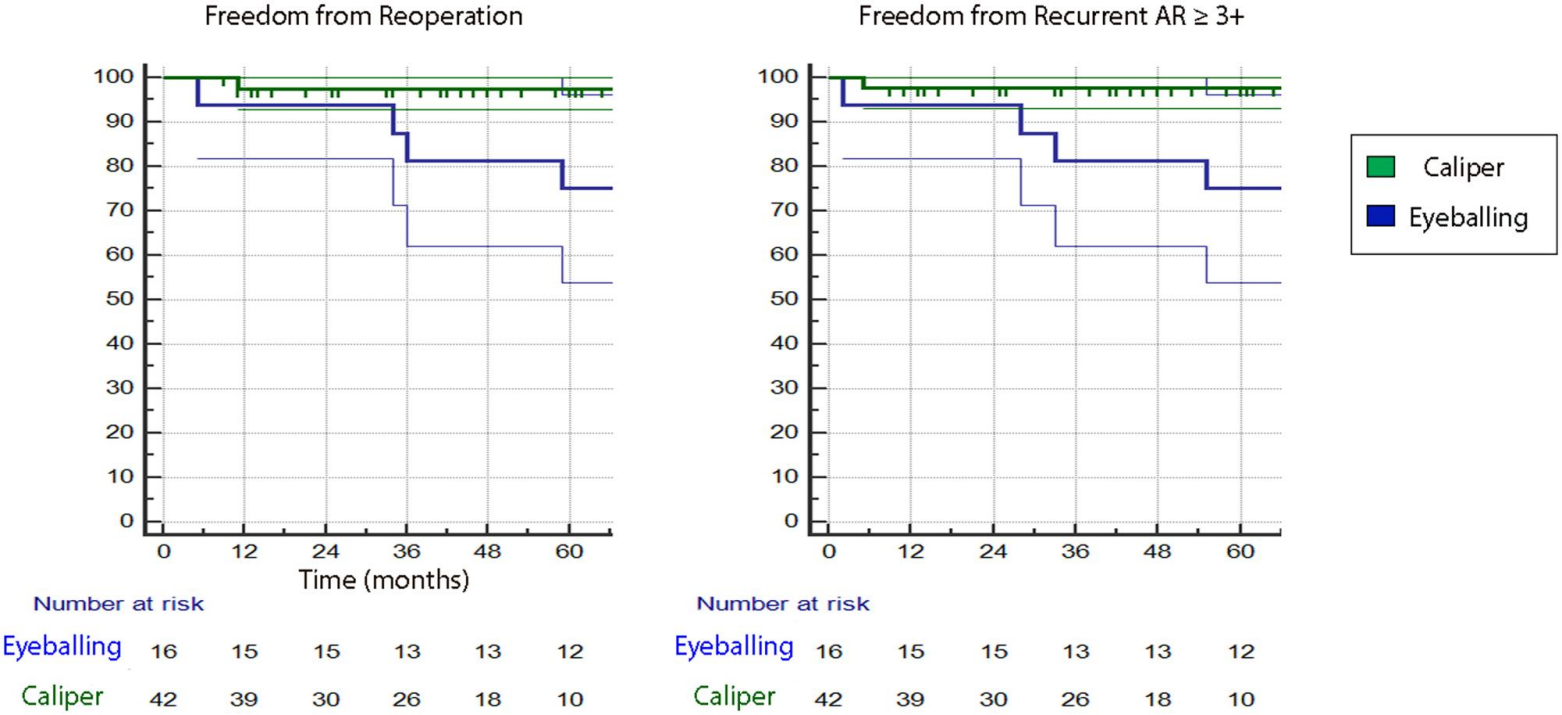
Kaplan–Meier curves showing freedom from reoperation and from recurrent aortic regurgitation (AR) ≥ 3+, comparing the use of caliper vs eyeballing in leaflet assessment.

At the beginning of 2018, we introduced the systematic cusps evaluation with the caliper to guide the cusp repair; for this reason, we also analysed our outcome, dividing the patient’s cohort according to the routine use of the caliper. We found that six out of eight patients with recurrent AR belong to the NO-CALIPER group, and only two to the CALIPER group, with a difference in 5-year freedom from recurrent AR of 97.6 ± 2.3% (caliper) vs 75.0 ± 10.8% (no-caliper), *P* = 0.16, and in 5-year freedom from reoperation 97.6 ± 2.4% (caliper) vs 75.0 ± 10.8% (no-caliper), *P*=0.03 (Fig. 3).

We also compared patients’ groups according to baseline annulus diameter, the analysis showed that, beyond the use of caliper descripted above, the baseline annulus dilatation (above 28 mm) was associated with a reduction in freedom from recurrent AR (40.0 ± 21.9% vs 84.6 ± 8.1%; *P*=0.001) and freedom from reoperation (40.0 ± 21.9% vs 91.3 ± 5.0%; *P*=0.004).

**Figure 4:**
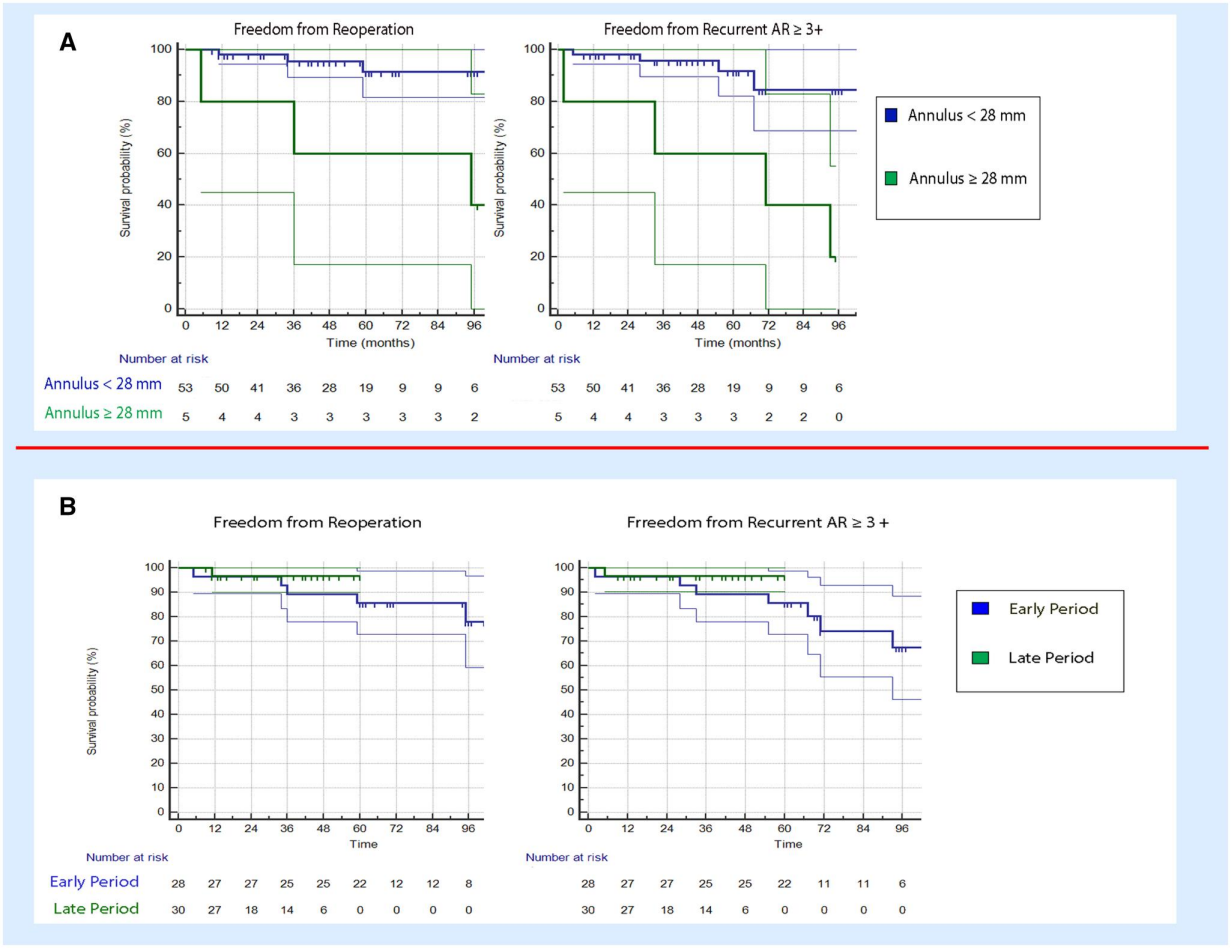
(**A**) Kaplan–Meier curves showing freedom from reoperation and from recurrent aortic regurgitation (AR) ≥ 3+, comparing native annulus <28 mm vs ≥28 mm. (**B**) Kaplan–Meier curves showing freedom from reoperation and from recurrent AR ≥ 3+, comparing early experience vs late experience.

The learning curve period clearly showed a difference between the early and the late experience, although not significant (freedom from recurrent AR 64.0 (3.0) months vs 67.7 (2.2) months; freedom from reoperation 64.8 (2.7) months vs 67.9 (2.1) months), but could be also affected by the introduction of the caliper (Fig. 4).

## DISCUSSION

In the last years, there has been a marked increase in the techniques of sparing or repairing the aortic valve. Avoidance of valve replacement is particularly appealing in the young patient population where the use of anticoagulant and its related morbidity is definitely undesirable [22].

In this respect, experience in the remodelling and reimplantation valve sparing techniques has played a significant role in advancing our understanding of the geometry, physiology and dynamics of the aortic root [23].

Particularly, the importance of re-establishing a normal geometry and dimension of the aortic annulus has appeared as the most important single step for a long-last optimal result. The reimplantation procedure is generally preferred for its intrinsic ability to reduce and stabilize the annulus diameter, while the addition of some form of annuloplasty is becoming more frequent for those surgeons who prefer the remodelling technique [24].

The importance of annuloplasty in all cases of aortic valve repair, even in the absence of a dilated root, is now clear. Annuloplasty reconstructs geometry, restores the coaptation and reduces cusp stress and stabilizes annular dimension over time. In this regard the sub-commissural annuloplasty (or Cabrol stitch) used to decrease the annulus diameter by virtually abolishing the inter-leaflet triangle has failed the test of time for its inability to prevent annular redilatation [25]. Furthermore, it eliminates the physiological role of the inter-leaflet triangles (i.e., the ability to transmit the ventricular pressure up to the top of the commissures).

At present, two different approaches to aortic valve annuloplasty are being increasingly used. On one side, the internal approach where the aortic annuloplasty is achieved by an internal rigid ring [26, 27]. While proponents of the internal rigid ring claim a consistent and predictable re-shaping of the annulus to facilitate leaflet plasty and to create a more durable condition, there are still concerns regarding the potential inflammatory reaction, the risk of leaflet restriction, contact and abrasion, haemolysis or thromboembolism.

An alternative option is the external approach, in which a flexible ring or a suture is positioned externally around the annulus. While the flexible ring is fixed by a series of sutures positioned below the leaflet inside out of the aortic annulus in a manner similar to that used for the reimplantation technique [28], the suture is simply circumferentially placed from outside. Despite being a fast and simple procedure, the external suture might have the potential risk of dehiscence and less accurate annular remodelling. On the other hand, a deep external dissection is an important prerequisite to place a ring around the VBR. Often, the surrounding structures such as the left and right atrium or the ventricular septum can be attached to the aortic wall at a level higher than the aortic annulus and need to be carefully dissected out. We lack sufficient scientific data to establish the superiority of one technique over the other.

Because of our group’s experience with the reimplantation technique and deep external root dissection, we have so far preferred the external approach by placement of a tailored Teflon ring (8 to 9 cm), aiming to obtain a desired internal annular diameter of about 21–23 mm.

Schäfers *et al.* have designed a caliper that facilitates easy and reproducible measurement of cusp dimensions as height difference, called “effective height” [29]. In order to standardize the assessment of leaflet prolapse and cusp configuration, the use of this caliper is recommended (using an effective height of 9–10 mm as a reference). If one cusp is found to be prolapsing (having an ‘effective height’ inferior to the other two cusps), the simple shortening of the leaflet’s free margin, by central plication, can eliminate the tissue redundancy and normalize cusp geometry. We demonstrated that the routine use of caliper has contributed to an improvement of the outcomes in terms of freedom from recurrent AR. As more data are collected, we will be able to better understand how to achieve long-term durability of a repaired aortic valve in a safe, reproducible and standardized fashion. It is possible in BAV with no or mildly dilated root that a more aggressive approach of root replacement might help in a better anatomical and symmetrical BAV repair.

It should be noted that the incidence of recurrent valve regurgitation at mid-term follow-up could be influenced by the patients’ adherence to therapy and to meticulous cardiologic follow-up for prevention of cardiac risk factors [30], especially in those with no early failure of the repair.

### Limitations

The single-centre nature of the study and its retrospective design are the main limitations. Although the aim of this study was to represent a 10-year experience, only 37% of patients reached the 5-year follow-up, furthermore we used a non-standard approach, using telephonic interviews for those patients, without complication, which did not require an echocardiography evaluation at our centre. The small number of patients, their characteristics and the technique features, with many variables, reduce the generalizability of the study. Secondary prevention after surgery may be a key factor in determining the recurrence of AR; adherence to therapy and cardiological follow-up were not assessed in this study.

Ideal multicentre study can allow different techniques comparison and the control of confounding factors.

## CONCLUSIONS

In conclusion, the aortic valve repair with external annuloplasty with a Teflon ring, appears a safe procedure with high overall survival (97.4 ± 2.6%), freedom from endocarditis (98.3 ± 1.7%) and freedom from thromboembolism (100%) at mid-term follow-up. The recurrence of AR and subsequent reoperation appears to be related to three major factors: the presence of dilated aortic annulus (>28 mm), eyeballing cusp repair (instead of routine caliper-tailored repair), learning curve. Larger studies with longer follow-up may be helpful to clarify the significance of each other’s failure predictors.

## Supplementary Material

ivaf146_Supplementary_Data

## Data Availability

The data underlying this article will be shared on reasonable request to the corresponding author.

## ABBREVIATIONS

AR: Aortic regurgitation
BAV: Bicuspid aortic valve
eH: Effective height
STJ: Sino-tubular junction
VBR: Virtual basal ring
